# Synopsis of the World Health Organization Guidelines on Childhood and Adolescent Obesity Management

**DOI:** 10.1002/gin2.70094

**Published:** 2026-10-27

**Authors:** Maria Nieves Garcia‐Casal, Hector Pardo‐Hernandez

**Affiliations:** ^1^ Department of Nutrition and Food Safety World Health Organization Geneva Switzerland

**Keywords:** adolescents, children, obesity management

## Abstract

Childhood and adolescent obesity is a complex, multifactorial condition resulting in long‐term health issues alongside diminished quality of life, educational attainment and overall wellbeing. The World Health Organization (WHO) prioritised the development of two guidelines on people‐centred, integrated management and care of (1) children and (2) adolescents with obesity. Key topics included dietary, physical activity, and behaviour changing interventions, multimodal approaches, digital health interventions, pharmacotherapy and bariatric surgery. WHO strongly recommends a multidisciplinary approach to obesity management delivered and supervised by health workers, including dietary, physical activity and behaviour changing interventions delivered individually or as part of multimodal interventions. WHO conditionally recommends digital health interventions under strict parental supervision on content and recreational screen time. Pharmacotherapy and bariatric surgery are not recommended for children. These interventions may be considered for adolescents after close consideration of physical and psychological development and should be prescribed and delivered in a specialised multidisciplinary setting. All WHO regions and countries are eligible for the implementation of these guidelines. They should be integrated into a continuum‐of‐care approach within existing child and adolescent health programmes, with strong intersectoral collaboration to enhance sustainability and scale‐up. The development of these guidelines followed a living methodology; future updates will consider emerging evidence on interventions, indicators, dosages, values and preferences, human rights, equity and gender considerations, intervention coverage, impact of new technologies and social media and the need for recommendations for specific populations.

## Summary

- **What We Already Know**
  ∘ Childhood and adolescent obesity is a complex, multifactorial condition resulting in diminished physical and mental health, alongside lower quality of life, educational attainment, and overall well‐being.
  ∘ Close to 170 million children and adolescents worldwide have obesity.
- **What This Adds to What Was Known?**
  ∘ WHO prioritised the development of two guidelines on people‐centred, integrated management and care of (1) children and (2) adolescents with obesity.
  ∘ These guidelines provide adoptable and adaptable, clear, practice‐ and science‐informed global clinical and public health recommendations for improved health, functioning and reduced obesity‐associated disability.
- **What Is the Implication and What Should Change Now?**
  ∘ Obesity management should consider the gender, health status, cultural context, interests, values and preferences, and cognitive capabilities of the child or adolescent; be age‐appropriate and culturally sensitive; and include support from parents and/or caregivers.
  ∘ Obesity management should move beyond a singular focus on weight loss and also prioritise improvement in overall health, functioning and physical and mental well‐being.

**Abbreviations**
BMI: body mass index
GDG: Guideline Development Group
GRC: World Health Organization Guideline Review Committee
WHO: World Health Organization

## 1 Background

Childhood and adolescent obesity is a complex, multifactorial condition driven by environmental, psychosocial and genetic influences, with further contributions from medications, diseases, or disability [1, 2]. Close to 160 million children and adolescents worldwide have obesity [3, 4, 5].

The consequences of childhood and adolescent obesity are profound, encompassing increased risks of cardiovascular disease, type II diabetes, respiratory conditions, cancers, musculoskeletal disorders, and mental health conditions, alongside diminished quality of life, educational attainment, and overall well‐being. Childhood and adolescent obesity contributes to obesity and poor health persisiting into adulthood, resulting in significant costs and strains to the health system [6, 7].

While current World Health Organization (WHO) and UNICEF recommendations have prioritised obesity prevention, there is a need for normative guidance to safeguard health across the life course, reduce early onset of noncommunicable diseases, and interrupt the persistence of obesity into adulthood [6].

WHO prioritised the development of two guidelines on people‐centred, integrated management and care of (1) children and (2) adolescents with obesity. These guidelines [8, 9] carefully considered state‐of‐the‐art evidence on obesity management interventions and the values and preferences of children and adolescents with obesity and their parents or caregivers. The guidelines acknowledged the inclusion of everyone, regardless of gender, age, race, ethnicity, sexual orientation, religion or disability status, while committing to justice, equality, impartiality and fairness. Box 1 summarises the main characteristics of these guidelines.

Box 1 **Scope, population and target users.**

**Table:** 

Scope	To provide practice‐ and science‐informed clinical and public health recommendations on the integrated management of children adolescents with obesity for improved health, functioning and reduced obesity‐associated disability, using a primary health care approach.
Questions	Prioritised topics included health services that are routinely offered in the primary health care setting: Dietary interventions Physical activity, sleep hygiene and sedentary behaviours Behaviour changing interventions Multimodal approaches Digital health interventions The guidelines also considered that interventions may require referral to secondary or tertiary care: Pharmacotherapy Bariatric surgery
Population	Children less than 10 years of age Adolescents 10–19 years of age
Target users	Decision‐makers and public health policymakers Health workers, including providers at the primary care level Integrated health programme managers and health system managers Non‐governmental and professional societies The general public and parents or caregivers of children and adolescents with obesity

## 2 Full Guideline Version

The full WHO guidelines version can be accessed:

- WHO guidelines on the integrated management of obesity in adolescents (https://iris.who.int/handle/10665/386830).
- WHO guidelines on the integrated management of obesity in children (https://iris.who.int/handle/10665/386828).

## 3 Recommendations

WHO strongly recommends a multidisciplinary approach for obesity management, including dietary, physical activity and behaviour‐changing interventions, delivered individually or as part of multimodal interventions, and supervised by health workers [8, 9]. As presented on Box 2, the choice of intervention should consider the characteristics of the patient, family and cultural context.

Box 2 **Summary of recommendations on dietary, physical activity, behaviour changing and multimodal interventions.**

**Table:** 

Children	Structured dietary, physical activity, behaviour‐changing, and multimodal interventions are recommended: Including parents and/or caregivers Considering the health status and the physical and cognitive capabilities of the child or adolescent Incorporating the interests and the cultural context of the child or adolescent and of parents and/or caregivers	Strong recommendations in favour
Adolescents

Overall, WHO promotes healthy diets as a cornerstone for preventing malnutrition in all its forms, emphasising dietary diversity, high intake of fruits, vegetables, and legumes, and the moderation of saturated and trans fats, salt and free sugars [10]. Physical activity may include organised sport activities, free play, or movement as part of daily activities. WHO recommends that children and adolescents meet the required physical activity levels for their age as stated in the 2020 WHO guidelines on physical activity and sedentary behaviour [11, 12].

The new guidelines also addressed behaviour‐changing interventions, considering the type of action they promote as educational/informational, persuasive, normative/structural, environmental and policy‐based interventions.

WHO conditionally recommends digital health interventions under strict parental supervision for content and recreational screen time (Box 3) [8, 9]. Digital health interventions may include active video games, exergames, exercise activity applications, pedometers or accelerometers [8, 9].

Box 3 **Summary of recommendations on digital health interventions.**

**Table:** 

Children	Digital health interventions may be recommended as part of an integrated approach to obesity management and under parental supervision for content and recreational screen time. Digital health interventions leading to physical activity and behavioural weight‐related modifications are desirable while limiting sedentary screen time Video games, exergames, exercise activity mobile applications, pedometers or accelerometers, and other similar interventions may have potential for increasing physical activity	Conditional recommendation in favour
Adolescents

Pharmacotherapy and bariatric surgery are not recommended for children [8]. These interventions may be considered for adolescents after close consideration of physical and psychological development (Box 4) and should be prescribed and delivered in a specialised multidisciplinary setting [9].

Box 4 **Summary of recommendations on pharmacotherapy and bariatric surgery.**

**Table:** 

Children	Pharmacotherapy and bariatric surgery are not recommended for children	Strong recommendation against
Adolescents	Pharmacotherapy and bariatric surgery delivered in a specialised multidisciplinary setting may be recommended for adolescents with obesity, but only: After a structured and supervised multimodal lifestyle modification has failed to achieve satisfactory results, and… After an assessment of pubertal development, final adult height, and psychological stability, and… Monitoring for potential adverse events	Conditional recommendations in favour

## 4 Methods

These guidelines followed the WHO Handbook for Guideline Development [13]. The WHO Obesity Steering Committee, led by the Department of Nutrition and Food Safety, oversaw the development process. The WHO Obesity Steering Committee participated in all stages of the process, including defining the scope of work, advising on the two Guideline Development Groups (GDG) composition, one GDG for children and one for adolescents, formulating questions and outcomes, overseeing evidence retrieval and synthesis, managing conflicts of interest, supervising external review and WHO Guideline Review Committee (GRC) approval, and finalising publication and dissemination [8, 9].

The WHO Obesity Steering Committee developed an initial set of questions and outcomes for public notice and comment, following consultation with regional and country offices and relevant WHO programmes. The results of these consultations were sent to the respective GDGs, which discussed findings and proposed a list of questions and outcomes for guideline development.

The GDGs brought together a group of experts with experience in obesity management, representing all WHO regions, gender, age and country income level. Systematic review teams, supported by the WHO Secretariat, conducted comprehensive evidence retrieval, appraisal, and synthesis following the WHO, Cochrane and GRADE methodological guidance [8, 9].

GDG meetings included GDG members, systematic review teams, topic‐specific experts, observers, and the WHO Secretariat. All GDG members, review teams, observers and external reviewers completed declarations of interest and confidentiality forms. Any relevant, actual or perceived conflicts of interest were declared in advance and at the beginning of each meeting. External reviewers critically appraised the guidelines to ensure relevance, rigour and applicability. The final versions of the guidelines were sent to the WHO GRC for final review and ratification [8, 9].

## 5 Evidence Summary

Most obesity management evidence in children and adolescents concentrates on anthropometric outcomes, especially body mass index (BMI), while offering limited insight into physical and mental well‐being, quality of life or obesity‐related disability. To address these gaps, WHO‐commissioned reviews that deliberately assessed a broader set of outcomes, harms and long‐term effects, and gender, equity, human rights and disability considerations.

Evidence searches followed standard systematic review methodologies. Besides structured PICO questions, background questions were developed to capture updated knowledge on obesity related topics not suited for formal systematic review formats or not conceived for issuing recommendations, including clinical assessment, follow‐up, referral pathways, provider competencies and the role of genetics, sex/gender, disability and mental health in obesity management [8, 9].

### 5.1 Cochrane Systematic Reviews

WHO commissioned 10 Cochrane systematic reviews on dietary interventions, physical activity, multimodal, digital health and pharmacological interventions for obesity management in children and adolescents. Overall, the quality of the evidence was low to very low. Outcomes focused mostly on anthropometric measures, with limited benefits across interventions. Length of follow‐up was short, generally 12 months or less.

### 5.2 Reviews on Values and Preferences

WHO commissioned seven reviews on values and preferences, specifically on obesity and body shape; obesity services and interventions; diet and physical activity; behaviour changing interventions; pharmacotherapy; bariatric surgery; and interaction with health workers.

### 5.3 Other Evidence Synthesis

WHO commissioned 17 additional reviews published in two special issues in the peer‐reviewed journals *Annals of the New York Academy of Sciences* and *Obesity Reviews*. These reviews focused on obesity causes, pathophysiological mechanisms and management approaches. They also assessed the role of bariatric surgery as well as key competencies of health workers in obesity management.

## 6 Evidence to Decision

Besides evidence on effects, decision‐making was informed by evidence on other considerations, which facilitated consensus and drafting of recommendations. These considerations included the balance between benefits and harms, values and preferences, costs, cost‐effectiveness, equity considerations, acceptability and feasibility of the interventions.

Obesity management interventions are likely to incur costs to families, which may affect adherence. Costs will depend on the specific requirements of an intervention, including diet composition, physical activity settings and equipment, health workers involved, the need for transportation or for time off work to attend scheduled appointments [8, 9].

Overall, dietary, physical activity and behavioural interventions are considered cost‐effective because of their potential in weight management and the prevention of chronic diseases in the long term. However, evidence on cost effectiveness comes mostly from high‐income countries and from adults [8, 9].

The effect of obesity management interventions on equity is unclear, given that studies on the topic are scarce. However, people of low socio‐economic status, disadvantaged communities, and individuals living with disabilities or chronic diseases are less likely worldwide to have access to obesity management interventions [8, 9].

The acceptability and feasibility of obesity management interventions increase when interventions are practical, engaging and appropriate for age, gender and ethnicity. Children and adolescents with obesity place a high value on the involvement of peers and family members. Interactions with health workers improve when compassionate, people‐first, non‐judgmental interactions are established. Lack of time, transportation, or resources to cover costs plays a key role in limiting feasibility [8, 9].

## 7 Discussion, Implementation and Further Steps

These guidelines are intended for implementation across all WHO regions and countries, supporting national health policies, strategies and plans in setting priorities to protect population health. They should be integrated into a continuum‐of‐care approach within existing child and adolescent health programmes, with strong intersectoral collaboration to enhance sustainability and scale‐up [8, 9].

Cost and resource requirements may limit the practical implementation and uptake of obesity management interventions, particularly for populations already experiencing health disparities, including people with low socioeconomic status, historically marginalised and minoritised ethnic groups, girls and people with disabilities. Without deliberate efforts to address these barriers, unequal access to obesity management interventions may widen existing health inequities, highlighting the need for whole‐of‐society approaches that improve accessibility and participation among underserved groups.

These guidelines followed a living methodology. Guideline updates will consider emerging evidence on efficacy, changes in values and preferences, human rights, equity considerations, intervention coverage, the impact of new technologies and social media. The WHO Department of Nutrition and Food Safety will review new evidence to determine whether existing recommendations should be revised, ensuring updates are integrated, clearly marked, and shared through WHO channels [8, 9].

Regarding research gaps, there is a need for well‐designed and well‐reported randomised controlled trials on the benefits and potential adverse events of obesity management interventions in the long term, with an emphasis on outcomes that go beyond anthropometric measures and that include physical and mental health wellbeing and reduction of obesity‐associated disability. Studies from low‐ and middle‐income countries and from minoritised communities are also required. Research results are to be disaggregated by sex and gender, severity of obesity, age groups, and disabilities. Further studies should ascertain the cost‐effectiveness of interventions in children and adolescents in low‐ and middle‐income settings, with different time horizons, effectiveness over time, and comparisons [8, 9].

In sum, the WHO guidelines on obesity management in children and adolescents provide adoptable and adaptable, clear, practice‐ and science‐informed global clinical and public health recommendations for improved health, functioning and reduced obesity‐associated disability, using a primary health care approach. Recommendations can be integrated into child and adolescent health programmes, aligned with national guidance and cross‐sectoral initiatives, to support coordinated implementation, strengthen sustainability, inform policy and create opportunities for scaling up interventions.

## Author Contributions

**Maria Nieves Garcia‐Casal:** conceptualisation, writing review and editing. **Hector Pardo‐Hernandez:** writing review and editing. Both authors approved the final manuscript.

## Conflicts of Interest

The authors declare no conflicts of interest.

## Data Availability

The authors have nothing to report.
